# Understanding the Potential Benefits of Cannabidiol for Patients With Schizophrenia: A Narrative Review

**DOI:** 10.1093/schizbullopen/sgab053

**Published:** 2021-11-28

**Authors:** Garrison J B Dyck, Zaid H Maayah, Dean T Eurich, Jason R B Dyck

**Affiliations:** 1 Cardiovascular Research Centre, Department of Pediatrics, Faculty of Medicine and Dentistry, University of Alberta, Edmonton, AB, Canada; 2 School of Public Health, University of Alberta, Edmonton, AB, Canada

**Keywords:** schizophrenia, cannabis, THC, CBD

## Abstract

Research suggests that cannabis-derived delta-9-tetrahydrocannabinol can be linked to the worsening of psychosis and/or other symptoms of schizophrenia. However, studies have shown that another major cannabinoid found in cannabis, cannabidiol (CBD), may be a potential alternative or adjunctive treatment for psychosis and schizophrenia. As such, herein we review the relevant literature relating to the safety and efficacy of CBD treatment in patients with schizophrenia, including the effects of CBD in treating the positive, negative, and cognitive symptoms of the disorder, as well as the molecular mechanisms by which CBD can reduce schizophrenic symptoms. The potential utility of CBD for mitigating cannabis cravings and cannabis withdrawal in this patient population will also be reviewed. Lastly, the dosing, method of drug delivery, length of treatment, and adverse effects of CBD in patients with schizophrenia are discussed. Thus, the goal of this narrative review is to help clinicians and researchers better understand the risks and benefits of this potential therapy for this patient population.

## Introduction

Multiple studies support the conclusion that cannabis use is associated with the development of psychosis and schizophrenia.^1–3^ For example, a robust longitudinal study in Sweden found that adolescent cannabis users had a 2.4 times higher chance of receiving a diagnosis of schizophrenia than those who had not used cannabis in adolescence.^2^ Similar studies have also shown a compelling link between cannabis use and the development of schizophrenia.^1,3^ Furthermore, the overwhelming majority of studies that interrogate the specific actions of individual cannabinoids suggest that delta-9-tetrahydrocannabinol (THC) is the main component of cannabis responsible for any worsening of psychosis and/or other symptoms of schizophrenia.^4,5^ With that being said, it is important to acknowledge that cannabis is a complex plant with hundreds of other active compounds, some of which may have opposite effects to THC in patients with schizophrenia.^6,7^ Indeed, evidence suggests that certain cannabinoids, such as cannabidiol (CBD), are potentially beneficial in the treatment of schizophrenia,^7^ and thus may be an alternative or adjunctive therapy for this patient population. Moreover, CBD may also have an advantage over existing treatments, as CBD is associated with fewer side effects than currently used dopaminergic antipsychotics.^8–12^ Thus, given the potential benefits of CBD in schizophrenia, a better understanding of this molecule is warranted.

Previous reviews provide a substantial amount of information about the beneficial effects of CBD for the treatment of patients with psychosis and/or schizophrenia.^13–22^ However, some of these reviews focused on discussing selected clinical studies on the basis of statistical analysis.^13–15^ In addition, others reviewed a possible role of CBD for treating addiction, cognitive dysfunction, and psychosis related to substance (eg, cannabis) use disorders^17–20^ or neurodegenerative disorders.^20^ To expand on these reviews, we have chosen to provide an update on the most recent advances made in the field, including a review of the evidence for the utility of CBD in treating the positive, negative, and cognitive symptoms of schizophrenia, as well as for treating cannabis cravings and cannabis withdrawal in patients with schizophrenia and a co-occurring cannabis use disorder (CUD) and/or cannabis dependence. We will also include an up-to-date discussion of the dose, method of drug delivery, length of treatment, and adverse effects of CBD, as well as the molecular mechanisms by which CBD can reduce schizophrenic symptoms. Thus, the goals of this narrative review are to: (1) comprehensively examine the current evidence regarding the effects of CBD in clinical studies involving patients with schizophrenia, and (2) discuss how this evidence may foster future research on compounds like CBD that have the potential to provide improved treatment and quality of life to those with the disorder.

### Overview of CBD

In 1940, CBD was the first identified nonpsychoactive cannabinoid in hemp^23^ and was then subsequently shown to be a nonpsychoactive components of *Cannabis sativa*.^24^ Since then, CBD has been found to possess multiple pharmacological actions such as analgesic, anti-inflammatory, anticonvulsant, and antioxidant effects, without causing the typical adverse effects of THC.^25,26^ Of interest, the pharmacological effect of CBD seems to be mediated via a cannabinoid receptor-independent mechanism, such as activation of the 5-hydroxytryptamine 1A (5-HT1A) receptor or suppression of a G-protein-coupled receptor 55 (GPR55).^27,28^ Moreover, based on clinical trials involving CBD-rich extracts, the FDA recently approved CBD for the management of treatment-resistant epilepsy such as Lennox-Gastaut syndrome and Dravet syndrome, indicating its safety and efficacy in this patient population.^29,30^ This recent advancement has not only demonstrated that CBD has the potential to therapeutically alter brain activity^31^ but it has also paved the way for considerations of CBD for the treatment of other neurological and mental health disorders, such as schizophrenia.^32^

## Methods

A literature search for English articles from 1970 to 2021 was conducted using Pubmed, Clinicaltrials.gov, and Google Scholar. Studies involving the use of CBD as intervention for schizophrenia and psychosis were selected using the terms “cannabidiol,” “CBD,” “schizophrenia,” “psychosis,” “psychotic symptoms.”

## Summary of Findings

### Clinical Evidence of the Potential Benefits of CBD in the Treatment of Schizophrenia

The efficacy of CBD as a therapy for the treatment of symptoms associated with schizophrenia was first reported in a clinical trial published in 2012.^8^ This randomized, double-blinded, parallel group, active-controlled study demonstrated the therapeutic effect of CBD as a monotherapy in the treatment of schizophrenia using the Positive and Negative Syndrome Scale: (PANSS) and Brief Psychiatric Rating Scale (BPRS) to assess patient reported changes in the progression of schizophrenia from baseline.^8^ In total, 39 patients with schizophrenia received either 800 mg/d oral gelatin capsule of CBD (*n* = 19) or the standard antipsychotic, amisulpride (*n* = 20) for 4 weeks. Notably, patients receiving CBD or amisulpride showed a statistically significant reduction of PANSS and BPRS for positive and negative symptoms of schizophrenia over the baseline.^8^ Of importance, the improvement in psychotic symptoms observed in CBD patients was similar to those who received amisulpride, suggesting that CBD is a promising candidate for the treatment of schizophrenia with comparable efficacy to the standard treatment.^8^ Interestingly, although the treatment with amisulpride was associated with typical side effects of antipsychotic drugs including motor disturbance, extrapyramidal symptoms, higher serum level of prolactin and weight gain, CBD was well tolerated and none of the aforementioned adverse events were reported in patients administered CBD. Thus, these findings suggest that CBD has a more favorable safety profile than amisulpride^8^ and provide evidence that CBD is equally efficacious as amisulpride for patients with schizophrenia.

Given the fact that: (1) at least 80% of people with schizophrenia suffer from cognitive impairment,^33^ and (2) the standard antipsychotic therapies have limited effects on cognitive performance in patients with schizophrenia,^34^ identifying new antipsychotic agents that can help reduce the cognitive impairment associated with schizophrenia is extremely important. To test if CBD causes cognitive improvements in people with schizophrenia, a total of 42 patients with schizophrenia were recruited in a randomized, double-blind, active-controlled, parallel group study (CBD-CT1; ClinicalTrials.gov identifier: NCT00628290) that assessed the efficacy of CBD vs amisulpride in improving neurocognitive performance in patients with acute schizophrenia.^9^ In this trial, patients received either 800 mg of an oral gelatin capsule of CBD per day (*n* = 21) or amisulpride (*n* = 21), over 4 weeks.^9^ Using 10 different neurocognitive performance tests, patients receiving oral CBD or amisulpride demonstrated a significant improvement in visual memory, processing speed, visuomotor coordination, and sustained attention over the baseline, with similar efficacy, suggesting that CBD is as efficacious as the standard antipsychotic in ameliorating cognitive impairments in patients with schizophrenia.^9^ In addition, previous research has demonstrated increased tolerability of CBD compared to amisulpride.^8^ Taken together, it is possible that CBD is a better treatment for cognitive impairments associated with schizophrenia than amisulpride. However, it is difficult to interpret the efficacy of CBD and amisulpride based on this trial due to the lack of a placebo group and the small sample size.^9^ In addition, the 4-week treatment duration is likely too short for the study of a chronic disease like schizophrenia.^9^ Thus, while the CBD-CT1 trial suggests an overall positive effect of CBD on neurocognitive function in those with schizophrenia,^9^ the findings need to be confirmed in a larger, more appropriately designed randomized controlled trial of patients with the disorder.

In addition to the CBD-CT1 trial, a multicenter, exploratory, prospective, randomized, parallel group, double-blind, placebo-controlled study was performed to assess the efficacy and safety of CBD as an adjuvant therapy for psychotic symptoms and cognitive impairments often associated with schizophrenia.^10^ In total, 88 subjects with schizophrenia received either an oral solution of CBD treatment (1000 mg/d, *n* = 43) or placebo (*n* = 45) and were assessed at baseline and at 6 weeks.^10^ Using PANSS as a primary endpoint for psychotic symptoms, patients receiving CBD showed a statistically significant reduction of PANSS for positive psychotic symptoms of schizophrenia, compared to the placebo group.^10^ In addition, while the difference was not statistically significant, patients treated with CBD demonstrated greater cognitive and motor speed performance compared to patients in the placebo group, as measured using the Brief Assessment of Cognition in Schizophrenia (BACS) composite score as a secondary endpoint. Thus, these findings suggest that CBD may be effective in treating psychotic symptoms and cognitive impairments associated with schizophrenia.^10^ Furthermore, although some patients in this trial experienced minor adverse effects such as headache, somnolence, abdominal pain, nausea, and diarrhea, CBD was well-tolerated overall.^10^ Together, the study confirms the potential efficacy and tolerability of CBD in patients with schizophrenia.^10^

#### The Efficacy and Safety of Low and Moderate Doses of CBD

Since lower doses of CBD are associated with low or even negligible side effects,^35^ 2 clinical studies investigated the efficacy and safety of low and moderate doses of CBD in patients with schizophrenia.^36,37^ The first trial was a 3 parallel-arm, double-blind, placebo control study to investigate the effect of CBD on cognitive dysfunction associated with schizophrenia.^36^ In this study, patients with schizophrenia (*n* = 28) received a single dose of 300 mg (*n* = 9) or 600 mg oral gelatin capsule of CBD (*n* = 9) or placebo (*n* = 10). Using the Stroop Colour Word Test as a primary endpoint for cognitive performance, patients receiving a low or a moderate dose of CBD did not demonstrate a significant improvement over the placebo group, suggesting that low and moderate doses of CBD are not efficacious in improving cognitive performance in those with schizophrenia.^36^ However, further study is warranted, as the trial was relatively small.

While the cognitive impairment associated with schizophrenia was not improved by a low or a moderate dose of oral CBD in the previous study,^36^ the possibility that moderate oral dose of CBD (ie, 600 mg/d) may reduce the positive and negative psychotic symptoms in patients with schizophrenia could not be entirely ruled out. Thus, a recent study examining the effects of a fixed, moderate dose of CBD in the treatment of the psychotic symptoms associated with schizophrenia was performed.^37^ This randomized, double-blind, placebo-controlled, add-on, parallel group study was conducted in 36 patients with schizophrenia.^37^ The patients with schizophrenia received either 600 mg of oral gelatin capsule of CBD (*n* = 18) or placebo (*n* = 18) daily for 6 weeks.^37^ Using PANSS and MATRICS Consensus Cognitive Battery (MCCB) to assess patients’ reported change in the progression of schizophrenia from baseline, treatment with 600 mg of CBD did not show a beneficial effect on psychotic symptoms and cognitive impairment on the primary outcome,^37^ suggesting that this dose of CBD (ie, 600 mg/d) is not effective in the treatment of positive and negative symptoms of schizophrenia. However, CBD was well tolerated with no worsening of mood, movement, psychotic symptoms, or cognitive function.^37^

Although the reasons for the discrepancy between the aforementioned studies^36,37^ and the other large clinical trials^8–10^ discussed above are unknown, it is likely that the differences may be attributed to the study size and possibly the dose and treatment duration. For instance, both the Boggs et al and Hallak et al studies used lower doses of CBD (600 mg/d and either 300 or 600 mg/d, respectively)^36,37^ over a short period of time^36^ (6 weeks and a single dose, respectively) compared to the other large clinical trials.^8–10^ Thus, given the fact that CBD has very low bioavailability due to its extensive first pass metabolism in the liver,^38^ it is possible that the lack of efficacy of CBD in the treatment of schizophrenia in these studies^36,37^ can be attributed to the variability of the oral bioavailability of CBD caused by the use of lower doses of CBD.^38^ In contrast, the efficacy of CBD reported in the larger trials^8–10^ could be related to the longer treatment duration and the larger dose used, which would cause a relatively high serum level of CBD. This interpretation of the findings suggests that a high oral dose of CBD is required to achieve better outcomes in the treatment of schizophrenia. In support of this, a recent systemic review of CBD dosing in patients with different types of diseases suggests that higher doses of CBD have a tendency to improved therapeutic outcomes compared to lower doses.^39^ Importantly, it should be noted that many of the patient reported outcome measures in the studies discussed are associated with a relatively high degree of variability between patients. As a result, it is unclear if the smaller studies, which often use lower doses and shorter periods of study, were sufficiently powered to allow identification of differences across the study groups.

#### The Efficacy and Safety of CBD in Small-Scale Open-Label Studies

Many other small case studies have investigated the efficacy of CBD in the treatment of symptoms associated with schizophrenia.^11,40–42^ For instance, the first study involved a patient with acute exacerbated schizophrenia treated with 1500 mg of CBD daily over 4 weeks.^40^ Notably, the patient showed a significant reduction of BPRS for positive symptoms of schizophrenia over the baseline, with no adverse effects.^40^ The second case study was conducted on a patient who was already resistant to the standard antipsychotic therapies and was admitted to the hospital after a suicide attempt.^11^ While the patient showed a modest response when treated with 500 mg of CBD twice daily for 7 weeks, an improvement in PANSS for positive and negative psychotic symptoms of schizophrenia was observed following an increase in CBD to 750 mg twice daily for 8 months, suggesting that high dose of CBD and long treatment duration may be required in the management of treatment-resistant schizophrenia.^11^ However, though CBD was well tolerated, a mild transient hand tremor was reported.^11^ In contrast to the previous study,^11^ 40 mg/ day of CBD (with the dose being increased every 5 days up to 1280 mg/d over 30 days) did not have any beneficial effects in a small case series trial in 3 patients with treatment-resistant schizophrenia.^41^ Importantly, the authors interpreted this finding to suggest that the lack of efficacy of CBD is credited to the insufficient dose and short treatment duration.^41^ Similarly, a clinically significant improvement in patients treated with 800 mg/d of CBD as an adjunctive therapy for 6 months was noted in a case report study of a patient with treatment-resistant anxiety and psychosis, further strengthening the notion that positive outcomes may be only achieved with the use of higher doses of CBD over a longer treatment duration.^42^

#### Ongoing Clinical Trials

There are at least 2 ongoing clinical trials investigating the efficacy of CBD in the treatment of symptoms associated with schizophrenia (ClinicalTrials.gov identifier: NCT02504151) and (ClinicalTrials.gov identifier: NCT02926859). One trial is a randomized, double-blind, 2-period crossover trial examining the effect of CBD on early acute psychosis in patients with schizophrenia (*n* = 72) (NCT02504151). Subjects in period 1 were randomized to receive 800 mg of CBD daily for 4 weeks, followed by a placebo for 2 weeks (ie, washout period), and then a placebo for a period of 4 weeks, under a double-blind condition (NCT02504151). In contrast, subjects in period 2 were randomized to receive a placebo for a period of 4 weeks, followed by a placebo for another 2 weeks (ie, washout period), and then 800 mg of CBD daily for 4 weeks (NCT02504151). As a primary endpoint for psychotic symptoms, the investigators plan to use PANSS and the Clinical Global Impression of Severity scale (CGI) for positive and negative symptoms of schizophrenia, whereas Patient Assessment of Own Functioning Inventory (PAOFI) and the Quality-of-Life Scale (QLS) will be used to test the effect of CBD on functioning as a secondary endpoint (NCT02504151). The estimated completion date of this study is December 2021 (NCT02504151). The second study is a large, multicenter, double-blind, 2-arm, placebo control, randomized add-on trial investigating the effect of CBD on early remitted patients with schizophrenia (NCT02926859). In this study, patients with schizophrenia (*n* = 180) will receive either 800 mg of CBD daily as add-on to standard pharmacological treatment or placebo over 26 weeks (NCT02926859). The estimated completion date of this study is March 2023 (NCT02926859). Both studies will likely help to further elucidate the safety and efficacy of CBD in the treatment of schizophrenia (table 1).

**Table 1.** The Effect of CBD on Schizophrenia-Related Symptoms in Clinical Studies

Study Population	Study Design	CBD Dose	Effect	Ref
Patients with schizophrenia (*n* = 39)	Randomized, double-blinded, parallel group, active-controlled study	800 mg/d oral gelatin capsule of CBD or amisulpride for 4 weeks	Reduction of PANSS and BPRS for positive and negative symptoms of schizophrenia over the baseline	^8^
Patients with schizophrenia (*n* = 42)	Randomized, double-blind, active-controlled, parallel group study	800 mg/d oral gelatin capsule of CBD or amisulpride for 4 weeks	An improvement in visual memory, processing speed, visuomotor coordination, and sustained attention	^9^
Patients with chronic schizophrenia (*n* = 88)	Multicenter, exploratory, prospective, randomized, parallel group, double-blind, placebo-controlled study	1000 mg/d oral solution of CBD for 6 weeks	Reduction of PANSS for positive psychotic symptoms of schizophrenia	^10^
Patients with schizophrenia (*n* = 28)	Three parallel-arm, double-blind, placebo control study	Single dose of 300 or 600 mg oral gelatin capsule of CBD	No significant improvement in cognitive performance	^36^
Patients with chronic schizophrenia (*n* = 36)	Randomized, double-blind, placebo-controlled, add-on, parallel group study	600 mg/d oral gelatin capsule of CBD for 6 weeks	No significant reduction in PANSS and MCCB for psychotic symptoms and cognitive performance	^37^
Patients with acute exacerbated schizophrenia (*n* = 1)	Open-label trial	1500 mg/d oral gelatin capsule of CBD for 4 weeks	Reduction in BPRS for positive symptoms of schizophrenia	^40^
Patients with treatment-resistant schizophrenia (*n* = 1)	Open-label trial	750 mg of CBD twice daily for 8 months	Reduction in PANSS for positive and negative psychotic symptoms of schizophrenia	^11^
Patients with treatment-resistant schizophrenia (*n* = 3)	Open-label case series trial	40 mg/ day with the dose being increased every 5 days up to 1280 mg/d over 30 days	No significant reduction in psychotic symptoms	^41^
Patients with anxiety and positive psychotic symptoms (*n* = 1)	Open-label trial	800 mg/d of CBD for 6 months	A clinically significant improvement in anxiety and positive psychotic symptoms	^42^
Patients with early psychosis (*n* = 13)	Experimental single dose clinical trials	Single oral dose of 600 mg oral gelatin capsule of CBD	Reduction in psychotic symptoms and an increase in hippocampal glutamate	^43^
Patients with clinical high risk (CHR) of psychosis (*n* = 33)	Experimental single dose clinical trials	Single oral dose of 600 mg oral gelatin capsule of CBD	CBD normalized the abnormal function in the striatum, midbrain, and hippocampus regions of the brain	^44^
Patients with CHR of psychosis (*n* = 33)	Experimental single dose clinical trials	Single oral dose of 600 mg oral gelatin capsule of CBD	Reduction in the abnormal activation in the left insula/parietal operculum	^45^
Patients with chronic schizophrenia (*n* = 72)	Randomized, double-blind, 2-period crossover trial	800 mg/d of CBD (4 + 2 + 4 weeks)	Ongoing clinical trial	NCT02504151
Patients with chronic schizophrenia (*n* = 180)	Multicenter, double-blind, 2-arm, placebo control, randomized add-on trial	800 mg/d of CBD for 26 weeks	Ongoing clinical trial	NCT02926859

*Note*: BPRS, Brief Psychiatric Rating Scale; CBD, cannabidiol; CHR, clinical high risk; MCCB, MATRICS Consensus Cognitive Battery; PANSS, Positive and Negative Syndrome Scale.

### Potential Mechanisms of the Antipsychotic Effects of CBD

**Fig. 1.**
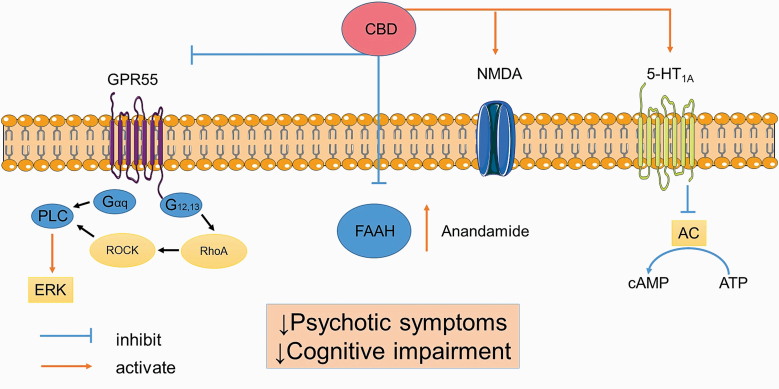
Potential mechanisms of action of CBD. CBD activates 5-HT1A and glutamate receptor (NMDA), inhibits FAAH enzyme, elevates serum anandamide, and blocks GPR55 receptor to control symptoms associated with schizophrenia. *Note*: 5-HT1A, 5-hydroxytryptamine 1A; AC, adenylate cyclase; cAMP, cyclic adenosine monophosphate; CBD, cannabidiol; ERK, extracellular signal-regulated kinase; FAAH, fatty acid amide hydrolase; GPR55, G-protein-coupled receptor 55; MCCB, MATRICS Consensus Cognitive Battery; NMDA, *N*-methyl-d-aspartate; PLC, phospholipase C; RhoA, Ras homolog family member A; ROCK, Rho-associated protein kinase. The image was modified from Servier Medical Art, licensed under a Creative Common Attribution 3.0 Generic License, https://smart.servier.com/.

The mechanisms by which CBD controls symptoms associated with schizophrenia were investigated in a study which found that 1 month of CBD treatment led to an improvement in psychotic symptoms.^8^ Of interest, in this study, the antipsychotic effect of CBD in patients with schizophrenia was associated with higher serum levels of endogenous anandamide over the baseline.^8^ Moreover, a reduction in schizophrenic symptoms in those who received CBD was significantly correlated with the serum level of anandamide, suggesting that endogenous anandamide might contribute to the antipsychotic effect of CBD.^8^ Furthermore, since fatty acid amide hydrolase (FAAH) is a key enzyme for the catabolism of endogenous anandamide,^46^ the authors also investigated the effect of CBD on FAAH in vitro in rat brain membranes.^8^ From this research, the authors found that CBD significantly reduced the enzymatic activity of FAAH, suggesting that the antipsychotic effect of CBD may be due, in part, to an impairment in the activity of FAAH and a subsequent elevation of serum anandamide^8^ (figure 1).

Contrary to the previous study,^8^ the CBD-CT1 trial demonstrated that the improvement in neurocognitive performance in patients with schizophrenia treated with CBD was not significantly correlated with the serum level of anandamide.^9^ This suggests that the mechanism by which CBD exerts its beneficial effects on cognitive function seems to be independent of the anandamide signaling and involves additional pathways. Given that: (1) activation of 5-HT1A receptor improves neurocognitive performance in schizophrenia,^47^ and (2) CBD causes allosteric activation of 5-HT1A receptor,^26,48^ it is possible that CBD may improve the cognitive dysfunction associated with schizophrenia via activation of the 5-HT1A receptor. In support of this, a recent preclinical study demonstrated that the beneficial effect of CBD on cognitive function in an animal model of schizophrenia was completely blocked by a 5-HT1A receptor antagonist, suggesting a 5-HT1A-dependent mechanism^12^ (figure 1).

Additional molecular targets of CBD that have been described include suppression of a GPR55.^27^ GPR55 is an orphan class A putative novel cannabinoid receptor that utilizes Gαq and Gα12/13 proteins to signal through phospholipase C and extracellular signal-regulated kinases (ERK1/2) phosphorylation.^49^ Of interest, the GPR55 recpetor is abundant and widely expressed throughout the CNS (ie, the hippocampus, forebrain, striatum, cerebellum, and cortex) and is involved in the pathogenesis of several CNS diseases including schizophrenia, epilepsy, and Alzheimer’s disease, via promotion of neuroinflammation and increased neuronal excitability.^50–52^ Thus, CBD may improve the psychotic symptoms and cognitive impairments associated with schizophrenia by blocking the GPR55 receptor and causing a subsequent reduction in neuroinflammation and neuronal excitability^27,51–53^ (figure 1).

In addition, numerous preclinical studies indicate that CBD significantly reduces psychotic symptoms induced by glutamate receptor blockers like ketamine by increasing the brain level of glutamate and potentiating the *N*-methyl-d-aspartate receptor.^54–57^ Thus, given the fact that schizophrenia is characterized as a hypoglutamatergic disorder,^58,59^ it is possible that CBD may reduce psychotic symptoms associated with schizophrenia via activation of glutamate receptors. To test this hypothesis, a recent randomized, placebo-controlled, double-blind, within-subject crossover design, repeated-measures study investigated the role of a single dose of CBD (600 mg) in hippocampal glutamate in patients with early psychosis (table 1). In this study, an improvement in PANSS for both positive and negative psychotic symptoms was observed in those with early psychosis treated with CBD.^43^ This improvement in patients administered CBD also coincided with a significant increase in hippocampal glutamate when compared to subjects administered a placebo.^43^ Additionally, the increase in hippocampal glutamate in the CBD treated group was significantly correlated with a reduction in psychotics symptoms in those with early psychosis. Thus, increased hippocampal glutamate might contribute in the antipsychotic effect of CBD.^43^

**Fig. 2.**
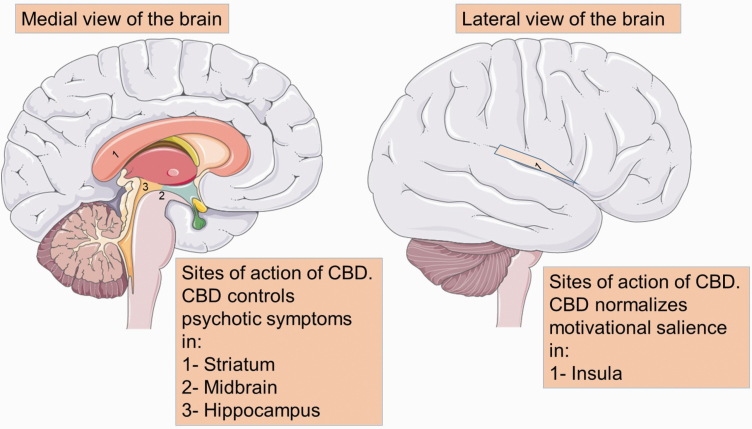
Sites of action of CBD. Psychotic symptoms are believed to arise from overactivity of neurons located in the hippocampus, which in turn releases dopamine to the midbrain and the striatum. Dopaminergic neurons in the midbrain further promulgate dopamine through a projection to the prefrontal cortex. Additionally, dopaminergic neuron terminals of the insula, originating in the striatum, are believed to influence dopamine neuronal reactivity and are critical for motivational salience and motor response. CBD controls psychotic symptoms in the striatum, midbrain, and hippocampus, as well as by normalizing motivational salience and motor response in the insula. *Note*: CBD, cannabidiol. The image was modified from Servier Medical Art, licensed under a Creative Common Attribution 3.0 Generic License, https://smart.servier.com/.

The site of antipsychotic action of CBD has also been investigated in a double-blind, randomized, parallel-arm, placebo-controlled trial in 33 patients with a clinical high risk (CHR) of psychosis and 19 healthy controls.^44^ This study aimed to assess the neurocognitive mechanism and the site of action of CBD in patients with CHR of psychosis, using functional magnetic resonance imaging (fMRI), while patients participated in a verbal learning task.^44^ A total of 33 patients with CHR of psychosis received either a single oral dose of 600 mg oral gelatin capsule of CBD (*n* = 16) or placebo (*n* = 17).^44^ Of note, healthy control subjects (*n* = 19) received neither placebo nor CBD.^44^ Using fMRI, it was found that CHR of psychosis patients who received placebo exhibited abnormal function in the striatum, midbrain, and hippocampus, when compared to the healthy control subjects.^44^ Of interest, administration of a single dose of CBD partially, but significantly, normalized the abnormal function in the aforementioned regions of the brain, suggesting that the striatum, midbrain, and hippocampus are main brain regions that underlie the beneficial effects of CBD on psychosis^44^ (figure 2).

Similar to the previous study,^44^ another randomized, double-blind, parallel-arm, placebo-controlled study also investigated the neurocognitive mechanism and the site of action of CBD in 33 patients with CHR of psychosis and 19 healthy controls.^45^ The main purpose of this study was to assess the effect of CBD on the motivational salience associated with psychosis, using fMRI, while patients participated in the monetary incentive delay task.^45^ Motivational salience was examined using reward processing and loss anticipation,^45^ both of which are known to be dysfunctional during psychosis.^60^ Patients who received placebo demonstrated premature action initiation (ie, false-start) and a significant abnormal activation in the left insula/parietal operculum, relative to the healthy subjects.^45^ Motivational salience and positive psychotic symptoms were also significantly correlated with the abnormal activation in the insula, suggesting that this part of the brain is strongly implicated in the pathophysiology of psychosis onset.^45^ Of interest, administration of a single dose of CBD (600 mg) significantly reduced the abnormal activation in the left insula/parietal operculum and slowed down the overall reaction time, suggesting that the antipsychotic effect of CBD may be mediated by normalizing motivational salience and moderating motor response in the insula^45^ (figure 2).

In light of the information described above, unlike a classical antipsychotic agent, the beneficial effect of CBD on schizophrenia seems to be independent of antagonizing dopamine receptors, suggesting that CBD represents an additional novel class of antipsychotic treatment with a unique mechanism of action. Indeed, activation of 5-HT1A and glutamate receptors,^12,43^ downregulation of FAAH enzyme,^8^ elevation of serum anandamide,^8^ and a suppression of the GPR55 receptor and neuroinflammation^27,51–53^ in the medial temporal lobe^44^ and insula^45^ might be potential mechanisms of action of CBD in the treatment of psychotic symptoms and the cognitive impairment associated with schizophrenia (figures 1 and 2).

### The Potential Role of CBD in Reducing Cannabis Cravings and Cannabis Withdrawal in Patients With Schizophrenia and a Co-occurring CUD

Given the high rates of CUD among patients with schizophrenia,^15,61,62^ and the lack of existing pharmacotherapies for CUD,^63^ understanding the potential of new drug therapies for CUD and/or cannabis dependence in this patient population is imperative. Indeed, since CBD has a modulatory role in various neurotransmitter systems and neural pathways involved in addiction, it has been proposed that CBD may have the potential to help treat substance use disorders, including CUD, potentially by reducing cannabis cravings and cannabis withdrawal.^63^ However, despite this potential, studies investigating CBD treatment for CUD in patients with co-occurring schizophrenia are lacking.^15^ That being said, in the general CUD patient population, the efficacy and safety of CBD as a treatment for cannabis cravings and cannabis withdrawal, which are often associated with CUD, have been more thoroughly investigated.^17^

Four double-blind randomized placebo-controlled clinical trials in patients with cannabis dependence found mostly beneficial effects of a CBD/THC mixture (Sativex) in treating cannabis cravings and cannabis withdrawal symptoms.^63–65^ Specifically, in a double-blind randomized placebo-controlled clinical trials in 27 patients with cannabis dependence, patients were given a 12-week treatment of either self-titrated dosages of Sativex (up to 113 mg of THC/105 mg of CBD/d) or placebo, in combination with cognitive behavioral therapy.^63^ Cannabis cravings were assessed with the Marijuana Craving Questionnaire—Short Form (MCQ-SF), while cannabis withdrawal was assessed with the Marijuana Withdrawal Checklist (MWC).^63^ Results from the trial indicated that Sativex was not only well tolerated but was also associated with a significantly greater decrease in cannabis cravings, compared to placebo.^63^ However, no significant effects in abstinence rates were observed in the trial and Sativex did not significantly affect cannabis withdrawal, compared to placebo.^63^ In contrast, in a similar double-blind randomized placebo-controlled trial in 9 patients with cannabis dependence, patients were given an 8-week treatment of either self-titrated doses of Sativex (up to 108 mg THC/100 mg CBD), fixed doses of Sativex (108 mg THC/100 mg CBD), self-titrated placebo, or fixed doses of placebo.^65^ Although Sativex treatment did not significantly affect cannabis cravings, Sativex treatment did significantly reduce cannabis withdrawal.^65^ Of interest, the largest reduction in cannabis withdrawal symptoms was observed with high-fixed doses of Sativex (108 mg THC and 100 mg CBD).^65^ However, in a different trial, daily treatment of Sativex with up to 86.4 mg of THC and 80 mg of CBD was found to have only transient effects in reducing cannabis cravings and withdrawal in patients with cannabis dependence, with no significant effects at 28 days post Sativex treatment.^64^ Lastly, in another clinical trial in 128 participants with cannabis dependence who were given a 12-week treatment of either Sativex (86.4 mg THC and 80 mg CBD/d) or placebo, in combination with psychosocial support, Sativex treatment was well tolerated and resulted in a significant reduction in days of illicit cannabis use compared with placebo.^66^ Taken together, this evidence indicates potential benefits for the use of CBD/THC mixtures in the treatment of CUD in the general CUD population, mainly by reducing cannabis cravings and/or cannabis withdrawal.^17^ Therefore, given these results, it is possible that CBD/THC mixtures could be helpful in treating CUD in patients with schizophrenia. However, there are currently no studies investigating this type of mixed therapy in patients with both schizophrenia and a CUD. Additionally, given the aforementioned risks surrounding THC use in patients in schizophrenia,^1,3^ this type of therapy may prove to be unsafe in this specific patient population and thus needs to be further studied.

Given the risks of THC consumption in patients with schizophrenia, CBD alone (ie, no THC) would likely be a better treatment option for CUD in patients with this disorder.^1,3^ However, there are currently no well-controlled studies that investigate the effects of pure CBD treatment in patients with both schizophrenia and co-occurring CUD.^15^ That being said, the effects of pure CBD have been investigated in the general cannabis dependence patient population, albeit less extensively than for CBD/THC mixtures. For instance, evidence from 2 case reports suggests that CBD treatment may alleviate cannabis withdrawal symptoms^67^ and promote cannabis abstinence^68^ for certain patients with cannabis dependence. Furthermore, in an open-label clinical trial with 25 frequent cannabis users, participants were given 200 mg of CBD daily, without discontinuing their normal cannabis use.^69^ Results from this study indicated that CBD treatment was well tolerated and reduced psychotic-like symptoms and cognitive impairments post-treatment, when compared to baseline.^69^ Intriguingly, this study also found that plasma CBD concentrations were positively correlated with improvements in psychological and cognitive symptoms, and that the greatest improvements were seen in dependent users.^69^ Overall, the authors proposed that their results suggest that CBD may be effective as an adjunctive treatment in patients with cannabis dependence.^69^

Taken together, these studies provide some evidence that pure CBD may have beneficial effects in treating CUD in the general CUD patient population.^17^ However, given the absence of randomized clinical trials that investigate the effects of pure CBD in the treatment of CUD,^17^ the evidence for the beneficial effects of CBD treatment in the general CUD patient population is only preliminary. Furthermore, due to the lack of existing studies with patients with schizophrenia, it is currently unknown if these preliminarily beneficial effects of pure CBD treatment in the general CUD population would translate to patients with both schizophrenia and co-occurring CUD.^15^ However, given the promising preliminary results in the general CUD patient population,^69^ as well as the reported safety of CBD use in patients with schizophrenia,^8^ the utility of CBD in treating CUD (including cannabis withdrawal and cannabis cravings) in patients with schizophrenia surely warrants future study.^15^

## Discussion

### The Potential Benefits of CBD for Patients With Schizophrenia

Herein, we have reviewed how CBD has been shown to improve psychotic symptoms and cognitive impairments associated with schizophrenia in many but not all studies. In general, clinical studies involving different dosage forms of CBD (oral gelatin capsule or solution) yielded encouraging results for the prevention and/or treatment of positive and negative psychotic symptoms, as well as the cognitive impairments associated with schizophrenia.^8–11^ However, despite these findings, there are limitations regarding the evidence for the use of CBD as a treatment for schizophrenia. For example, most of the research previously discussed is only preliminary and has not involved large sample sizes.^8,9,11,44^ Furthermore, another barrier to fully understanding the utility of CBD treatment in patients with schizophrenia is the lack of knowledge regarding CBD treatments among certain subpopulations of patients with the disorder, such as those with a co-occurring CUD.^15^ Given that it has been proposed that people with schizophrenia may respond differently to CBD therapy depending on if they have CUD or not,^15^ as well as the preliminary evidence suggesting that CBD could improve CUD symptoms in the general CUD patient population,^69^ this is an area of study that warrants further research. Specifically, future researchers should consider designing trials that evaluate the possible differences between the effects of CBD in patients with schizophrenia that have CUD vs ones who do not. Lastly, randomized-controlled trials that asses the efficacy of CBD in treating cannabis cravings and cannabis withdrawal in patients with schizophrenia and a co-occurring CUD should also be a research priority.

Perhaps one of the largest challenges of CBD treatment in all patient groups is low oral bioavailability, due to its extensive first pass metabolism in the liver.^38^ Thus, it may not be surprising that higher doses of CBD have shown better therapeutic outcomes compared to lower doses.^8,9,11,39^ In fact, lower doses of CBD lack efficacy in the treatment of schizophrenia in some clinical studies,^36,37^ which further strengthens the notion that positive outcomes can only be achieved with the use of higher doses of CBD.^11,39^ However, the high oral doses of CBD that are needed for efficacy also increase the risk of adverse events. Indeed, adverse events such as headache, somnolence, abdominal pain, nausea, and diarrhea^10,35^ were noted in clinical studies using higher doses of CBD. In addition to the undesirable effects, given that schizophrenia is a chronic disease that would require daily use of CBD, cost is another major hurdle of the use of high oral doses of CBD.^70^ Thus, identifying a novel formulation or testing a new delivery system that would enable lower doses of CBD to be administered and be maintained at higher plasma levels could help solve many of the aforementioned issues. Nevertheless, despite these potential drawbacks, the preliminary evidence suggests promising beneficial effects of CBD in those with schizophrenia, which at the very least warrants future research on the utility, safety, and logistics of CBD-based therapies for this disorder.^8–11^

### Limitations

This narrative review did not follow a systematic literature search, meta-analysis, or data extraction approach. Also, there is no consideration of the quality of the presented evidence based on statistical analysis. As such, the interpretation of the data is based on a critical review by the authors of the article.

## Supplementary Material

sgab053_suppl_Supplementary_Material

## References

[1] Hall W, Degenhardt L. Cannabis use and the risk of developing a psychotic disorder. World Psychiatry. 2008;7(2):68–71. 18560513 10.1002/j.2051-5545.2008.tb00158.x PMC2424288

[2] Andréasson S, Allebeck P, Engström A, Rydberg U. Cannabis and schizophrenia. A longitudinal study of Swedish conscripts. Lancet. 1987;2(8574):1483–1486. 2892048 10.1016/s0140-6736(87)92620-1

[3] Hall W, Degenhardt L. Cannabis and the increased incidence and persistence of psychosis. BMJ. 2011;342:d719. 21363867 10.1136/bmj.d719

[4] Patel S, Khan S, Saipavankumar M, Hamid P. The association between cannabis use and schizophrenia: causative or curative? A systematic review. Cureus. 2020;12(7):e9309. 32839678 10.7759/cureus.9309 PMC7442038

[5] Hindley G, Beck K, Borgan F, et al. Psychiatric symptoms caused by cannabis constituents: a systematic review and meta-analysis. Lancet Psychiatry. 2020;7(4):344–353. 32197092 10.1016/S2215-0366(20)30074-2 PMC7738353

[6] Hagerty SL, Williams SL, Mittal VA, Hutchison KE. The cannabis conundrum: thinking outside the THC box. J Clin Pharmacol. 2015;55(8):839–841. 25855064 10.1002/jcph.511

[7] Davies C, Bhattacharyya S. Cannabidiol as a potential treatment for psychosis. Ther Adv Psychopharmacol. 2019;9:2045125319881916. 31741731 10.1177/2045125319881916 PMC6843725

[8] Leweke FM, Piomelli D, Pahlisch F, et al. Cannabidiol enhances anandamide signaling and alleviates psychotic symptoms of schizophrenia. Transl Psychiatry. 2012;2:e94. 22832859 10.1038/tp.2012.15 PMC3316151

[9] Leweke FM, Rohleder C, Gerth CW, Hellmich M, Pukrop R, Koethe D. Cannabidiol and amisulpride improve cognition in acute schizophrenia in an explorative, double-blind, active-controlled, randomized clinical trial. Front Pharmacol. 2021;12:614811. 33995015 10.3389/fphar.2021.614811 PMC8117353

[10] McGuire P, Robson P, Cubala WJ, et al. Cannabidiol (CBD) as an adjunctive therapy in schizophrenia: a multicenter randomized controlled trial. Am J Psychiatry. 2018;175(3):225–231. 29241357 10.1176/appi.ajp.2017.17030325

[11] Makiol C, Kluge M. Remission of severe, treatment-resistant schizophrenia following adjunctive cannabidiol. Aust N Z J Psychiatry. 2019;53(3):262–265. 30543310 10.1177/0004867418815982

[12] Rodrigues da Silva N, Gomes FV, Sonego AB, Silva NRD, Guimaraes FS. Cannabidiol attenuates behavioral changes in a rodent model of schizophrenia through 5-HT1A, but not CB1 and CB2 receptors. Pharmacol Res. 2020;156:104749. 32151683 10.1016/j.phrs.2020.104749

[13] Kopelli E, Samara M, Siargkas A, Goulas A, Papazisis G, Chourdakis M. The role of cannabidiol oil in schizophrenia treatment. A systematic review and meta-analysis. Psychiatry Res. 2020;291:113246. 32599446 10.1016/j.psychres.2020.113246

[14] Ghabrash MF, Coronado-Montoya S, Aoun J, et al. Cannabidiol for the treatment of psychosis among patients with schizophrenia and other primary psychotic disorders: a systematic review with a risk of bias assessment. Psychiatry Res. 2020;286:112890. 32126328 10.1016/j.psychres.2020.112890

[15] Ahmed S, Roth RM, Stanciu CN, Brunette MF. The impact of THC and CBD in schizophrenia: a systematic review. Front Psychiatry. 2021;12:694394. 34366924 10.3389/fpsyt.2021.694394 PMC8343183

[16] Schoevers J, Leweke JE, Leweke FM. Cannabidiol as a treatment option for schizophrenia: recent evidence and current studies. Curr Opin Psychiatry. 2020;33(3):185–191. 32073423 10.1097/YCO.0000000000000596

[17] Batalla A, Janssen H, Gangadin SS, Bossong MG. The potential of cannabidiol as a treatment for psychosis and addiction: who benefits most? A systematic review. J Clin Med. 2019;8(7):1058. 31330972 10.3390/jcm8071058 PMC6678854

[18] Bartoli F, Riboldi I, Bachi B, et al. Efficacy of cannabidiol for delta-9-tetrahydrocannabinol-induced psychotic symptoms, schizophrenia, and cannabis use disorders: a narrative review. J Clin Med. 2021;10(6):1303. 33810033 10.3390/jcm10061303 PMC8005219

[19] Chesney E, Oliver D, McGuire P. Cannabidiol (CBD) as a novel treatment in the early phases of psychosis. Psychopharmacology (Berl). 2021. 10.1007/s00213-021-05905-9 PMC9110455 34255100

[20] Osborne AL, Solowij N, Weston-Green K. A systematic review of the effect of cannabidiol on cognitive function: relevance to schizophrenia. Neurosci Biobehav Rev. 2017;72:310–324. 27884751 10.1016/j.neubiorev.2016.11.012

[21] Schubart CD, Sommer IE, Fusar-Poli P, de Witte L, Kahn RS, Boks MP. Cannabidiol as a potential treatment for psychosis. Eur Neuropsychopharmacol. 2014;24(1):51–64. 24309088 10.1016/j.euroneuro.2013.11.002

[22] Hahn B. The potential of cannabidiol treatment for cannabis users with recent-onset psychosis. Schizophr Bull. 2018;44(1):46–53. 29083450 10.1093/schbul/sbx105 PMC5768049

[23] Filer CN. Minnesota wild hemp: a crucial botanical source in early cannabinoid discovery. J Cannabis Res. 2020;2(1):25. 33526126 10.1186/s42238-020-00031-3 PMC7819329

[24] Robson PJ. Therapeutic potential of cannabinoid medicines. Drug Test Anal. 2014;6(1–2):24–30. 24006213 10.1002/dta.1529

[25] Morgan CJ, Schafer G, Freeman TP, Curran HV. Impact of cannabidiol on the acute memory and psychotomimetic effects of smoked cannabis: naturalistic study: naturalistic study [corrected]. Br J Psychiatry. 2010;197(4):285–290. 20884951 10.1192/bjp.bp.110.077503

[26] Maayah ZH, Takahara S, Ferdaoussi M, Dyck JRB. The molecular mechanisms that underpin the biological benefits of full-spectrum cannabis extract in the treatment of neuropathic pain and inflammation. Biochim Biophys Acta Mol Basis Dis. 2020;1866(7):165771. 32201189 10.1016/j.bbadis.2020.165771

[27] Devinsky O, Cilio MR, Cross H, et al. Cannabidiol: pharmacology and potential therapeutic role in epilepsy and other neuropsychiatric disorders. Epilepsia. 2014;55(6):791–802. 24854329 10.1111/epi.12631 PMC4707667

[28] Ibeas Bih C, Chen T, Nunn AV, Bazelot M, Dallas M, Whalley BJ. Molecular targets of cannabidiol in neurological disorders. Neurotherapeutics. 2015;12(4):699–730. 26264914 10.1007/s13311-015-0377-3 PMC4604182

[29] Chen JW, Borgelt LM, Blackmer AB. Epidiolex (cannabidiol): a new hope for patients with Dravet or Lennox-Gastaut syndromes. Ann Pharmacother. 2019;53(15):1060028018822124. 10.1177/1060028018822124 30616356

[30] Urits I, Borchart M, Hasegawa M, Kochanski J, Orhurhu V, Viswanath O. An update of current cannabis-based pharmaceuticals in pain medicine. Pain Ther. 2019;8(1):41–51. 30721403 10.1007/s40122-019-0114-4 PMC6514017

[31] Compton WM, Einstein EB. The need for evidence regarding cannabidiol. JAMA Netw Open. 2020;3(10):e2021067. 33057640 10.1001/jamanetworkopen.2020.21067

[32] Sholler DJ, Schoene L, Spindle TR. Therapeutic efficacy of cannabidiol (CBD): a review of the evidence from clinical trials and human laboratory studies. Curr Addict Rep. 2020;7(3):405–412. 33585159 10.1007/s40429-020-00326-8 PMC7880228

[33] Kremen WS, Seidman LJ, Faraone SV, Toomey R, Tsuang MT. The paradox of normal neuropsychological function in schizophrenia. J Abnorm Psychol. 2000;109(4):743–752. 11196000 10.1037//0021-843x.109.4.743

[34] Keefe RS, Bilder RM, Davis SM, et al.; CATIE Investigators; Neurocognitive Working Group. Neurocognitive effects of antipsychotic medications in patients with chronic schizophrenia in the CATIE Trial. Arch Gen Psychiatry. 2007;64(6):633–647. 17548746 10.1001/archpsyc.64.6.633

[35] Huestis MA, Solimini R, Pichini S, Pacifici R, Carlier J, Busardò FP. Cannabidiol adverse effects and toxicity. Curr Neuropharmacol. 2019;17(10):974–989. 31161980 10.2174/1570159X17666190603171901 PMC7052834

[36] Hallak JE, Machado-de-Sousa JP, Crippa JA, et al. Performance of schizophrenic patients in the Stroop Color Word Test and electrodermal responsiveness after acute administration of cannabidiol (CBD). Braz J Psychiatry. 2010;32(1):56–61. 20339735 10.1590/s1516-44462010000100011

[37] Boggs DL, Surti T, Gupta A, et al. The effects of cannabidiol (CBD) on cognition and symptoms in outpatients with chronic schizophrenia a randomized placebo controlled trial. Psychopharmacology (Berl). 2018;235(7):1923–1932. 29619533 10.1007/s00213-018-4885-9

[38] Gaston TE, Friedman D. Pharmacology of cannabinoids in the treatment of epilepsy. Epilepsy Behav. 2017;70(Pt B):313–318. 28087250 10.1016/j.yebeh.2016.11.016

[39] Millar SA, Stone NL, Bellman ZD, Yates AS, England TJ, O’Sullivan SE. A systematic review of cannabidiol dosing in clinical populations. Br J Clin Pharmacol. 2019;85(9):1888–1900. 31222854 10.1111/bcp.14038 PMC6710502

[40] Zuardi AW, Morais SL, Guimarães FS, Mechoulam R. Antipsychotic effect of cannabidiol. J Clin Psychiatry. 1995;56(10):485–486. 7559378

[41] Zuardi AW, Hallak JE, Dursun SM, et al. Cannabidiol monotherapy for treatment-resistant schizophrenia. J Psychopharmacol. 2006;20(5):683–686. 16401651 10.1177/0269881106060967

[42] Berger M, Li E, Amminger GP. Treatment of social anxiety disorder and attenuated psychotic symptoms with cannabidiol. BMJ Case Rep. 2020;13(10):e235307. 10.1136/bcr-2020-235307 PMC7542610 33028567

[43] O’Neill A, Annibale L, Blest-Hopley G, Wilson R, Giampietro V, Bhattacharyya S. Cannabidiol modulation of hippocampal glutamate in early psychosis. J Psychopharmacol. 2021;35(7):814–822. 33860709 10.1177/02698811211001107 PMC8278563

[44] Bhattacharyya S, Wilson R, Appiah-Kusi E, et al. Effect of cannabidiol on medial temporal, midbrain, and striatal dysfunction in people at clinical high risk of psychosis: a randomized clinical trial. JAMA Psychiatry. 2018;75(11):1107–1117. 30167644 10.1001/jamapsychiatry.2018.2309 PMC6248101

[45] Wilson R, Bossong MG, Appiah-Kusi E, et al. Cannabidiol attenuates insular dysfunction during motivational salience processing in subjects at clinical high risk for psychosis. Transl Psychiatry. 2019;9(1):203. 31439831 10.1038/s41398-019-0534-2 PMC6706374

[46] Ahn K, Johnson DS, Cravatt BF. Fatty acid amide hydrolase as a potential therapeutic target for the treatment of pain and CNS disorders. Expert Opin Drug Discov. 2009;4(7):763–784. 20544003 10.1517/17460440903018857 PMC2882713

[47] Meltzer HY, Sumiyoshi T. Does stimulation of 5-HT(1A) receptors improve cognition in schizophrenia? Behav Brain Res. 2008;195(1):98–102. 18707769 10.1016/j.bbr.2008.05.016

[48] Russo EB, Burnett A, Hall B, Parker KK. Agonistic properties of cannabidiol at 5-HT1a receptors. Neurochem Res. 2005;30(8):1037–1043. 16258853 10.1007/s11064-005-6978-1

[49] Ryberg E, Larsson N, Sjögren S, et al. The orphan receptor GPR55 is a novel cannabinoid receptor. Br J Pharmacol. 2007;152(7):1092–1101. 17876302 10.1038/sj.bjp.0707460 PMC2095107

[50] Gray RA, Whalley BJ. The proposed mechanisms of action of CBD in epilepsy. Epileptic Disord. 2020;22(S1):10–15. 32053110 10.1684/epd.2020.1135

[51] Khan AA, Shekh-Ahmad T, Khalil A, Walker MC, Ali AB. Cannabidiol exerts antiepileptic effects by restoring hippocampal interneuron functions in a temporal lobe epilepsy model. Br J Pharmacol. 2018;175(11):2097–2115. 29574880 10.1111/bph.14202 PMC5979781

[52] Na KS, Jung HY, Kim YK. The role of pro-inflammatory cytokines in the neuroinflammation and neurogenesis of schizophrenia. Prog Neuropsychopharmacol Biol Psychiatry. 2014;48:277–286. 23123365 10.1016/j.pnpbp.2012.10.022

[53] Mori MA, Meyer E, Soares LM, Milani H, Guimarães FS, de Oliveira RMW. Cannabidiol reduces neuroinflammation and promotes neuroplasticity and functional recovery after brain ischemia. Prog Neuropsychopharmacol Biol Psychiatry. 2017;75:94–105. 27889412 10.1016/j.pnpbp.2016.11.005

[54] Gomes FV, Issy AC, Ferreira FR, Viveros MP, Del Bel EA, Guimaraes FS. Cannabidiol attenuates sensorimotor gating disruption and molecular changes induced by chronic antagonism of NMDA receptors in mice. Int J Neuropsychopharmacol. 2014;18(5):1–10. 10.1093/ijnp/pyu041 PMC4376539 25618402

[55] Long LE, Malone DT, Taylor DA. Cannabidiol reverses MK-801-induced disruption of prepulse inhibition in mice. Neuropsychopharmacology. 2006;31(4):795–803. 16052245 10.1038/sj.npp.1300838

[56] Moreira FA, Guimarães FS. Cannabidiol inhibits the hyperlocomotion induced by psychotomimetic drugs in mice. Eur J Pharmacol. 2005;512(2–3):199–205. 15840405 10.1016/j.ejphar.2005.02.040

[57] Linge R, Jiménez-Sánchez L, Campa L, et al. Cannabidiol induces rapid-acting antidepressant-like effects and enhances cortical 5-HT/glutamate neurotransmission: role of 5-HT1A receptors. Neuropharmacology. 2016;103:16–26. 26711860 10.1016/j.neuropharm.2015.12.017

[58] Coyle JT. The glutamatergic dysfunction hypothesis for schizophrenia. Harv Rev Psychiatry. 1996;3(5):241–253. 9384954 10.3109/10673229609017192

[59] Cioffi CL. Modulation of NMDA receptor function as a treatment for schizophrenia. Bioorg Med Chem Lett. 2013;23(18):5034–5044. 23916256 10.1016/j.bmcl.2013.07.019

[60] Strauss GP, Waltz JA, Gold JM. A review of reward processing and motivational impairment in schizophrenia. Schizophr Bull. 2014;40(suppl 2):S107–S116. 24375459 10.1093/schbul/sbt197 PMC3934394

[61] Hasin DS, Kerridge BT, Saha TD, et al. Prevalence and correlates of DSM-5 cannabis use disorder, 2012–2013: findings from the National Epidemiologic Survey on Alcohol and Related Conditions-III. Am J Psychiatry. 2016;173(6):588–599. 26940807 10.1176/appi.ajp.2015.15070907 PMC5026387

[62] Koskinen J, Löhönen J, Koponen H, Isohanni M, Miettunen J. Rate of cannabis use disorders in clinical samples of patients with schizophrenia: a meta-analysis. Schizophr Bull. 2010;36(6):1115–1130. 19386576 10.1093/schbul/sbp031 PMC2963055

[63] Trigo JM, Soliman A, Quilty LC, et al. Nabiximols combined with motivational enhancement/cognitive behavioral therapy for the treatment of cannabis dependence: a pilot randomized clinical trial. PLoS One. 2018;13(1):e0190768. 29385147 10.1371/journal.pone.0190768 PMC5791962

[64] Allsop DJ, Copeland J, Lintzeris N, et al. Nabiximols as an agonist replacement therapy during cannabis withdrawal: a randomized clinical trial. JAMA Psychiatry. 2014;71(3):281–291. 24430917 10.1001/jamapsychiatry.2013.3947

[65] Trigo JM, Lagzdins D, Rehm J, et al. Effects of fixed or self-titrated dosages of Sativex on cannabis withdrawal and cravings. Drug Alcohol Depend. 2016;161:298–306. 26925704 10.1016/j.drugalcdep.2016.02.020 PMC4878903

[66] Lintzeris N, Bhardwaj A, Mills L, et al.; Agonist Replacement for Cannabis Dependence (ARCD) study group. Nabiximols for the treatment of cannabis dependence: a randomized clinical trial. JAMA Intern Med. 2019;179(9):1242–1253. 31305874 10.1001/jamainternmed.2019.1993 PMC6632121

[67] Crippa JA, Hallak JE, Machado-de-Sousa JP, et al. Cannabidiol for the treatment of cannabis withdrawal syndrome: a case report. J Clin Pharm Ther. 2013;38(2):162–164. 23095052 10.1111/jcpt.12018

[68] Shannon S, Opila-Lehman J. Cannabidiol oil for decreasing addictive use of marijuana: a case report. Integr Med (Encinitas). 2015;14(6):31–35. 26807069 PMC4718203

[69] Solowij N, Broyd SJ, Beale C, et al. Therapeutic effects of prolonged cannabidiol treatment on psychological symptoms and cognitive function in regular cannabis users: a pragmatic open-label clinical trial. Cannabis Cannabinoid Res. 2018;3(1):21–34. 29607408 10.1089/can.2017.0043 PMC5870061

[70] Elliott J, McCoy B, Clifford T, Potter BK, Wells GA, Coyle D. Economic evaluation of cannabinoid oil for Dravet syndrome: a cost-utility analysis. Pharmacoeconomics. 2020;38(9):971–980. 32406036 10.1007/s40273-020-00923-5

