# Supplementary material for: Understanding the Potential Benefits of Cannabidiol for Patients With Schizophrenia: A Narrative Review
Source: Schizophr Bull Open. 2021 Nov 28;3(1):sgab053. doi: 10.1093/schizbullopen/sgab053 (PMC11205871; doi:10.1093/schizbullopen/sgab053)
Supplement: sgab053_suppl_Supplementary_Material [file sgab053_suppl_Supplementary_Material.docx]

Supplementary material

**Clinical vignette:**

*The following vignette is published with our patient’s consent and was anonymized.*

Pierre, a 37 year-old man, is living with his wife and young child in the city of Lausanne, Switzerland. His parents divorced when he was 10 years old, which was experienced as traumatic. Before the divorce, Pierre and his family moved houses a lot. He developped the first symptoms of schizophrenia in fourth year of biology at the university (voices and persecutory delusions). Regarding his diagnosis, Pierre mentions, “It is as if I was fighting for many years against an ‘unknown evil’, and suddenly it had a name, schizophrenia”. While it was a relief to put a name on his suffering, the term schizophrenia also carried along frustration and despair. Pierre mentions that he knows he is lucky to have a family and to be able to take care of his kid when he is well enough; this brings meaning to his life. However, he mentions that “it is difficult to recover what one has lost”. He would like to start writing again but too often he doesn’t have the inspiration or the concentration to do so. His current biggest suffering is his lack of sense of purpose in society, “I wish I had an activity to do in society”.

Much later after the mapping session, Pierre admitted that he felt rambled and that therapy had touched some very intimate topics that he had not shared with anyone before. “Without realizing it, I revisited places inside of myself”. “I also realized the importance tea-rooms had in my life. I rarely go somewhere without stopping in a tea-room. I think this is a bit poor. When I look at this map, there should be more museums”.

The following indications are given to the patients to fill-in the diagram reported as supplementary Figure 1. 1): “Please choose 10 significant places (i.e. associated with positive or negative emotions), that are important to you in the present (including one favorite place and home)”. 2) “Locate these places on the first diagram; places located closer to the center represent places with stronger emotional bond or place attachment”. “Tag places if ambivalent feeling is associated with them”. 3) Repeat the same exercise on a diagram representing the “future” and one diagram representing the “past”.

**

**Caption supplementary Figure 1: Place attachment diagram**

*Tags in blue are the ones appearing in all diagrams*

It is interesting to notice whether there are places that are present in all three diagrams. Did new places appear or disappear in the “future” diagram? Are there any risks in going to the “future” diagram? What would you need to realize this “future” diagram?

Categories of places to look for are the following (modified from^1^): ﻿﻿(1) Home; (2) Social/ leisure (e.g. homes of friends/family, movie theaters, parks, forests, lakes/seashores, libraries, bookshops, museums), (3) Activities of daily living (e.g. shopping centers, restaurants, cafés, grocery stores), (4) Work/volunteer/educational, and (5) Physical/Mental health-related (e.g., mental health centers, hospitals)

In the place attachment diagram, the temporal dimension is present in two ways. First, place attachment itself has a temporal dimension embedded in its definition, as attachment usually develops over time. Second, the service user is asked to depict 3 diagrams (present, future, past) which represents a dynamic of change.

Places chosen by Pierre in the ***present*** diagram are his home, the *place de la Palud*, the *Payot bookshop*, a second-hand bookshop, Ouchy lake shore, his mother’s home, the mountain family chalet, his psychiatrist’s practice, his father’s home and *Cery hospital*. Without doubt, Pierre’s favorite place is the *place de la Palud* in Lausanne. He was born close to this square in the old town, had a good friend working in a jewelry shop close by; goes there for the farmers’ market twice a week. Goes there to drink a coffee or buy a pastry. He mentions “this place is part of my genes”. He describes a profound feeling of rootedness and belonging which goes back to his childhood pointing to the appearance of place attachment. Sense of belonging and restorative properties of this place are important in his recovery process.

He likes a lot the *Payot bookshop*; “I love spending hours there and look at books. I like to go there to reflect. Sometimes I stop at the philosophy section”. Pierre avoids places like his father’s house because he will feel trapped there. He usually avoids the forest; it can be source of restoration or anguish and often “trees are too close or too far away”. Regarding the *Ouchy* lake shore, Pierre mentions: “I look for the wind, water, the lake. There is a mixture of water and wind. In the past I have done a lot of sailing and when I’m there, I feel like going on a boat! I don’t have a boat, so I can’t do it. I like big open spaces. Get a feel of the natural elements makes me feel good. To walk near the water... I sometimes go with my kid but often I go alone”. Later, Pierre decided to do his sailing license: “Every year, this same idea comes back… but now, I have to do it, I like being on a boat, far away from the land, this brings me peace of mind”. He recognizes that being able to rent a boat at the local sailing school would give him access to a whole new space and geography.

When making the ***future*** diagram, Pierre mentioned “this is interesting, it is as if an atom liberated a photon, a change in energy”. “If I could make these changes, I would have a greater sense of security and I would feel more in peace”. On the place attachment diagram, he brought closer to the center, his father’s places as well as his home and added his son’s climbing activity. He would like to start climbing there. When asked about the risks to bring his father’s home closer to the center of the diagram, he mentioned that this was currently not possible because he often upsets Pierre and there is violence in his words. However, he says: “Being closer to my dad would allow me to be much more in peace and in serenity”. Regarding his home, he mentions, “To me, it is too square; and I’d rather live closer to the countryside”. Pierre also admits that one of his wishes would be to do some voluntary work in one of his favorite second-hand bookshops. The *place de la Palud* is of course also present in this diagram. “I should also be able to integrate the psychiatric hospital of *Cery* and give it some space even if it is not my wish.” In the ***past*** diagram, Pierre depicted 5 places: the *place de la Palud*, his father’s house, his mother’s house, and the farm house in the *Canton of Fribourg* and the swimming pool in Spain. “It’s cute to see to what a 5-year old’s life can be summarized to”. “I’m born near the *place de la Palud*, so this place was already important to me”. “Then we lived in this farm in Fribourg and also went back and forth to Spain for my father’s job. These are happy memories. When my parents separated, they each moved to another home”.

**Caption supplementary Figure 2: life-space network**

*Distance travelled are represented by edges and places are represented by nodes. Solid line represents current places and doted blue line represents future places.*

Here the patient is asked to position the 10 places (or more) chosen in the “place attachment diagram” on a map. Service users may have variable spatial capacities and may need help from the therapist. Then a picture is taken and the map is annotated and turned into a simple graph where places are nodes and routes are edges. The map can also be printed out again and location moved around, keeping in mind that all places form a network and moving one location may influence the emotional/physical distance to others.

Pierre has no difficulties in moving around the city. Of course, when he is too unwell, he will not drive but otherwise he walks, takes the bus or train and drives to farther locations. He likes to move around and especially walking: “walking is a great help; when I was unwell, I would walk a lot, always the same walks. When I walk, it calms my anxiety and calms my brain which turns in loops. When my kid was young, I would escape the noise.” Observing the map, Pierre mentions “My house should be in the center of the map, shouldn’t it?” When asked about the resources he is looking for in these places: “La *place de la Palud* represent my roots, the *Payot bookshop* represents knowledge for me”. “My house is affective; I like the contact with my family. My kid brings life and spontaneity to the house and drags me out of my lethargy”. “Tea-rooms are peaceful places to go and have a coffee; for me these are moments between two activities”. Pierre doesn’t go often to his father’s place. He rather takes walks with his father which gives him more freedom and less awkward moments, or moments where he feels caught in a double bind. “It gives movement to the discussion, it’s a neutral place, not his place not mine, we are nowhere”.

1. Townley G, Kloos B, Wright PA. Understanding the experience of place: Expanding methods to conceptualize and measure community integration of persons with serious mental illness. *Health Place*. 2009;15(2):520-531. doi:10.1016/j.healthplace.2008.08.011
